# Analysis of Cognitive Levels and Influencing Factors in Children with Obstructive Sleep Apnea

**DOI:** 10.3390/children11121428

**Published:** 2024-11-26

**Authors:** Jing Bi, Bo Yu, Guotong Zheng, Yangyan Yan, Yang Zhang, Xiaoyan Lin, Yiyuan Han, Chao Song

**Affiliations:** Children’s Hospital, Zhejiang University School of Medicine, National Clinical Research Center for Child Health, Hangzhou 310051, China; bijing-ent@zju.edu.cn (J.B.); 6520126@zju.edu.cn (B.Y.); 6522004@zju.edu.cn (G.Z.); 6513043@zju.edu.cn (Y.Y.); 6516022@zju.edu.cn (Y.Z.); 6522005@zju.edu.cn (X.L.); 6522219@zju.edu.cn (Y.H.)

**Keywords:** children, OSA, influencing factors, cognitive functioning

## Abstract

Background: Obstructive sleep apnea (OSA) in children is prevalent worldwide and affects their physiological, psychological, and cognitive functions. However, the research on OSA’s impact on children’s cognitive function remains inconclusive. This study aims to analyze the cognitive levels and influencing factors in children with OSA in a single-center study in China. Methods: We selected 110 children with OSA who visited the Department of Otorhinolaryngology, Head and Neck Surgery at the Children’s Hospital of Zhejiang University School of Medicine from March 2023 to April 2024. Cognitive function was assessed using the Wechsler Intelligence Scale for Children-Fourth Edition (WISC-IV), and data on the OAHI, BMI, lowest blood oxygen saturation, and tonsillar hypertrophy were collected. A correlation analysis was performed using SPSS Statistics 26.0. Results: The mean WISC-IV score of the children with OSA was 102.32, within the normal range. Gender and tonsillar grade had no significant effect on the cognitive levels. The BMI scores were significantly negatively correlated with verbal comprehension. The OAHI was weakly negatively correlated with perceptual reasoning but not with other cognitive dimensions. Conclusions: OSA may negatively affect specific cognitive domains in children, particularly perceptual reasoning and working memory. The BMI is a crucial factor influencing cognitive function in children with OSA. Future research should increase the sample size, include more variables, and conduct long-term follow-ups to comprehensively evaluate the influencing factors of OSA on children’s cognitive function.

## 1. Background

OSA is prevalent among children [1] with an incidence rate between 1% and 5% that increases with age. Studies show that the incidence rate of OSA in children aged 8 to 11 is 4.7%, with higher rates observed in Black children and preterm infants [2]. In Beijing, the prevalence among children aged 3 to 14 ranges from 5.90% to 8.08% [3]. Additionally, the rising rate of childhood obesity is associated with more severe OSA symptoms in children with higher body mass index (BMI) scores, potentially threatening their cognitive abilities [4]. Children affected by OSA may experience a range of physiological, psychological, and behavioral disorders, including distractibility, disruptive behavior, and impaired executive function [5,6]. However, the impact of OSA on children’s cognitive function remains a topic of debate in the academic community. Some studies suggest that mild to moderate OSA hypoventilation syndrome may adversely affect cognitive function in children under six years old but not significantly in those older than six [7]. Furthermore, Cardoso and colleagues analyzed studies published between 2002 and 2016 regarding the impact of OSA on children’s cognitive and behavioral abilities [8]. They found that, while OSA might lead to a decline in intellectual abilities, most affected children’s intellectual abilities remain within the normal range. Contrarily, other studies indicate that OSA can cause gray and white matter damage and functional abnormalities in the prefrontal cortex and hippocampus, significantly reducing cognitive and executive functions in children [9]. Moreover, a large-scale community cohort study [10] showed that, as the severity of sleep-disordered breathing increases, cognitive performance (including intellectual and language functions) deteriorates, with children with moderate to severe OSA being particularly affected. These findings suggest that, although there is evidence supporting the negative impact of sleep apnea on children’s cognitive function, further high-quality research is needed to validate and clarify the relationship. Therefore, exploring the specific factors influencing the cognitive levels of children with OSA becomes crucial [11,12]. Understanding these factors will not only help to explain the reasons for symptom variability but also lay the groundwork for defining preventive protocols and educational programs for children, parents, and caregivers. Such programs should focus on promoting healthy sleep habits, managing BMI, and early detection of OSA symptoms through targeted education and community interventions. This will provide a basis for developing effective interventions.

In exploring the factors affecting children’s cognitive levels, the BMI and obstructive apnea–hypopnea index (OAHI) values are important physiological indicators [13,14]. A prospective study by Hansen et al. found that higher BMI scores correlated with lower cognitive development scores in preschool children [15]. Additionally, a study using diffusion tensor imaging (DTI) investigated the impact of OSA severity on children’s white matter integrity and cognitive function, finding that greater OSA severity led to more significant white matter damage and cognitive decline [16]. Research by Khadra et al. on school-aged children indicated that sleep apnea reduces brain oxygen saturation, affecting cognitive function [17]. These studies reveal that BMI scores and OSA severity influence children’s cognitive levels. However, these physiological indicators are just the tip of the iceberg [18,19]: the factors affecting children’s cognitive levels extend far beyond these. A multidimensional and multidisciplinary research approach is necessary to thoroughly analyze and determine the accurate influencing factors.

To determine if OSA affects cognitive levels in children, we conducted a comprehensive study at a single center in China. We assessed cognitive function using the Wechsler Intelligence Scale for Children. The study also examined the correlations between the OAHI, lowest blood oxygen saturation, BMI, and tonsillar hypertrophy with cognitive outcomes.

## 2. Methods

### 2.1. Study Subjects

Children who visited the Department of Otolaryngology-Head and Neck Surgery at the Children’s Hospital, Zhejiang University School of Medicine, from March 2023 to April 2024, and had not undergone adenoidectomy and tonsillectomy, were recruited as study subjects. The inclusion criteria were as follows [20]: (1) presence of tonsillar hypertrophy and/or adenoid hypertrophy, accompanied by clinical symptoms such as snoring, mouth breathing, and apnea during sleep; (2) an obstructive apnea–hypopnea index (OAHI) greater than 1 episode per hour; (3) age range between 6 and 18 years. The exclusion criteria included the following: (1) presence of sleep disorders other than OSA; (2) central nervous system diseases or other neurological disorders that could impair cognitive function; (3) severe hearing or vision impairments that could influence cognitive assessment outcomes; (4) current use of medications affecting cognitive function; (5) previous adenoidectomy and/or tonsillectomy or cognitive assessment conducted within the past six months.

This study was approved by the Ethics Committee of the Children’s Hospital, Zhejiang University School of Medicine (Ethics Approval Number: 2024-IRB-0161-P-01).

### 2.2. Sample Size Calculation

This study examines 11 variables. According to the Kendall sample size estimation guideline [21], an appropriate sample size should be 5 to 10 times the number of variables. Consequently, the preliminary calculation suggests a sample size ranging from 55 to 110 participants. Accounting for a potential 10% rate of invalid responses, the adjusted sample size requirement increases to between 62 and 123 participants. In reality, this study included a sample size of 110 cases.

### 2.3. Research Methods

#### 2.3.1. General Clinical Data

In this study, we meticulously collected detailed clinical data on children diagnosed with OSA. Key variables included age, gender, disease duration, adenoid thickness to nasopharyngeal cavity width ratio (A/N ratio), tonsil grading, BMI, OAHI, and lowest blood oxygen saturation. We recorded the age and gender of all subjects with precision and calculated disease duration from the time of diagnosis. The A/N ratio was determined through imaging studies, while tonsil grading was assessed using the Brodsky scale [22]. BMI was calculated by dividing weight (kg) by the square of height (meters). According to the World Health Organization standards, calculate the Body Mass Index Standard Deviation Score (BMI Z-score) for children and classify their weight status into four categories: underweight (BMI Z-score < −1), normal weight (−1 ≤ BMI Z-score < 1), overweight (1 ≤ BMI Z-score < 2), and obesity (BMI Z-score ≥ 2). OAHI was measured using standard sleep monitoring equipment, and the lowest blood oxygen saturation was recorded during nighttime sleep with a pulse oximeter.

#### 2.3.2. Cognitive Level Assessment

This study evaluated the cognitive abilities of the enrolled children using the Chinese version of WISC-IV [23]. The Chinese WISC-IV is designed to assess the intelligence of children aged 6 to 16 years and 11 months. It includes 10 core subtests that measure four indices: Verbal Comprehension Index (VCI), Perceptual Reasoning Index (PRI), Working Memory Index (WMI), and Processing Speed Index (PSI). Intelligence is assessed based on the Full-Scale IQ (FSIQ), derived from these indices, as well as the individual index scores. The WISC-IV was standardized with a large, representative sample, and scores were statistically adjusted to achieve a mean of 100 and a standard deviation of 15, ensuring comparability across different age groups [24]. All WISC-IV tests in this study were administered by trained professionals who underwent standardized training before the study began to ensure consistency and reliability. This study used these norms to evaluate the children’s cognitive levels.

### 2.4. Statistical Analysis

The data were organized and analyzed using SPSS Statistics version 26.0. Normally distributed quantitative data were presented as mean ± standard deviation, while categorical data were expressed as frequencies or proportions. To compare means between two groups, an independent samples *t*-test was performed. For comparisons among more than two groups, a one-way ANOVA was conducted. The relationship between the children’s OAHI and cognitive levels was assessed using Pearson correlation analysis. Multiple linear regression analysis was employed to identify factors influencing the children’s cognitive levels. Statistical significance was set at *p* < 0.05.

## 3. Results

### 3.1. Descriptive Analysis

This study included an analysis of the demographic characteristics and physiological measurements of 110 children with OSA, as shown in Table 1. The average age of these children was 8.59 ± 2.00 years, with an age range of 6–14 years. Additionally, the average duration of the disease was 2.26 ± 1.64 years. Notably, the mean WISC-IV score was 102.32, indicating performance within the normal range, although there was significant individual variation.

### 3.2. Univariate Analysis

An analysis of the cognitive levels in children with OSA, considering both gender and tonsil grading, indicated that neither factor significantly affects cognitive levels. As shown in Table 2, the male children scored slightly higher on the total WISC-IV score than the female children; however, this difference was not statistically significant, suggesting a minimal impact of gender on cognitive function in children with OSA. Additionally, no significant differences were observed between genders regarding the VCI, PRI, WMI, or PSI. Furthermore, the tonsil grading results in Table 3 demonstrated no statistically significant differences in the total WISC-IV scores or individual cognitive sub-scores. These findings suggest that gender and tonsil grading show no statistically significant influence on the overall cognitive performance.

### 3.3. Correlation Analysis

Table 4 presents the results of the correlation analysis between various factors associated with OSA in children and different cognitive dimensions. Notably, age and BMI score were significantly correlated with several cognitive indicators. Age demonstrated a negative correlation with WISC-IV score (*p* < 0.05), particularly in the perceptual reasoning dimension (*p* < 0.01), indicating a decline in perceptual reasoning ability with increasing age. Similarly, BMI score showed a significant negative correlation with overall cognitive levels and VCI (*p* < 0.01). These findings suggest that a higher BMI may be linked to poorer cognitive performance, potentially due to metabolic disorders, inflammation, or other physiological mechanisms associated with obesity affecting brain function. Additionally, the duration of illness, A/N ratio, OAHI, and lowest blood oxygen saturation did not exhibit significant correlations with any cognitive dimensions. Although the OAHI had a weak negative correlation with perceptual reasoning, the relationship between the lowest blood oxygen saturation and any cognitive dimension was not statistically significant.

### 3.4. Regression Analysis

Using WISC-IV scores as the dependent variable, the variables that showed statistical significance in univariate analysis were selected as the independent variables for the multiple stepwise regression analysis. The variables that entered the regression equation are shown in Table 5.

## 4. Discussion

This study investigates the cognitive levels of children with OSA and the factors influencing these levels. The results show a weak negative correlation between the OAHI and perceptual reasoning, but polysomnographic variables did not significantly affect the total cognitive scale score. This suggests that OSA may have a limited impact on certain specific cognitive domains, while its overall effect on cognitive levels is less significant than initially hypothesized. Overall, the findings of this study support the view that OSA does not significantly affect the overall cognitive level of children, consistent with some previous studies [25,26]. However, our conclusions differ from some earlier research [27,28]. For instance, Bourke et al. conducted a cross-sectional study using overnight polysomnography (PSG) to categorize 137 children, followed by cognitive function assessments [29]. They found a positive correlation between the severity of OSA and cognitive impairment in children aged 7–12, especially in executive function and academic performance. Similarly, Daniel et al. studied 142 children and observed poor performance in working memory and processing speed tests regarding the WISC-IV among children with OSA, which could negatively impact academic achievement [30]. Moreover, Scott J et al. provided a comprehensive evaluation of intelligence, language, attention, memory, and executive function in children with OSA, reporting lower scores in FSIQ, VCI, and WMI [31]. These discrepancies highlight the need for caution in generalizing findings across studies and emphasize the importance of considering variations in sample sizes, study designs, and assessment tools. For example, Scott J et al. used the Differential Ability Scales and NEPSY cognitive assessment tools, which differ from the tools used in our study [31]. Therefore, future research should aim to increase sample sizes and utilize a broader range of assessment tools to more accurately evaluate the comprehensive impact of OSA on children’s cognitive levels. Enhancing the consistency of the research methodologies will help to clarify the specific cognitive domains affected by OSA and provide a clearer understanding of its overall impact.

Among the indicators for assessing the severity of OSA, the OAHI is weakly negatively correlated with the PRI. This finding aligns with other research. For instance, a study by Fan et al. demonstrated that severe OSA is significantly negatively correlated with cognitive impairments, particularly in areas related to the PRI, such as visuospatial skills, executive function, and verbal memory [32]. Moreover, neuroimaging studies, including one by Yu et al., have found that children with OSA exhibit reduced gray matter volume in those brain regions associated with perceptual reasoning, like the right middle frontal gyrus [33]. These gray matter volumes are also negatively correlated with the OAHI. Further analysis indicates that the OAHI primarily reflects the frequency of apneas and hypopneas during sleep. While the OAHI may not be directly related to overall WISC-IV scores, it has a more immediate impact on specific cognitive functions, such as perceptual reasoning. This may be because the intermittent hypoxemia and nocturnal oxygen desaturation caused by OSA can lead to brain hypoxia, affecting the structure and function of neural cells. For instance, the reduced low-frequency amplitude in the left angular gyrus and increased amplitude in the right insula are linked to this hypoxic state, which may cause abnormal neural activity in these brain regions, thereby impacting cognitive functions [34].

Research indicates that the cognitive level of children with OSA is influenced by various physiological factors, with BMI score being a significant one. BMI scores are significantly negatively correlated with WISC-IV scores, particularly in the VCI. An elevated BMI induces alterations in neural structures, including reductions in gray matter volume, which impact the critical brain regions that are responsible for language and emotion processing, thereby disrupting speech comprehension [35]. Specifically, the brain regions involved in decision-making and emotional regulation are adversely affected, hindering individuals’ ability to maintain attention and process language in complex linguistic contexts. Additionally, obesity is often accompanied by a chronic inflammatory state, with elevated levels of inflammatory cytokines such as IL-6. High IL-6 levels may affect the permeability of the blood–brain barrier, allowing more immune cells and inflammatory factors to enter the brain, exacerbating neuroinflammation and leading to cognitive decline [36]. This suggests the potential role of obesity management in improving cognitive function, which involves not only weight control but also considering its impact on inflammatory status and metabolic health [37].

This study has made significant progress in exploring the cognitive levels and influencing factors in children with OSA, but several limitations must be acknowledged. Firstly, the small sample size of this study limits the statistical power and generalizability of the results. For instance, in the multiple linear regression analysis, BMI score was found to be the only significant variable, further emphasizing its role in influencing cognitive levels. Although the Wechsler Intelligence Scale for Children, which includes normative data, was used as an assessment tool, enabling a comparison with the general population, these controls lack detailed information on factors such as BMI score. However, the lack of a specifically established control group to compare the impact of BMI score on children without OSA is a significant limitation. This limitation somewhat affects the validity of the findings and limits our ability to isolate the specific effects of BMI score on cognitive function in children with OSA compared to healthy children. Moreover, the study lacks long-term follow-up data, making it impossible to evaluate the long-term effects of OSA on cognitive levels. The study only assessed two OSA-related variables, the OAHI and blood oxygen saturation, neglecting other important physiological indicators, such as sleep architecture, breathing patterns, and heart rate variability, which could also affect cognitive function. Despite these limitations, the study found a negative correlation between the OAHI and perceptual reasoning, although this correlation was not statistically significant. Additionally, the absence of a control group limits the ability to draw definitive conclusions about the impact of OSA. These findings suggest that frequent sleep interruptions and hypoxic events may have a potential impact on specific cognitive functions. Future research should increase the sample size, establish control groups, and consider the lack of statistical significance when interpreting results. Long-term follow-ups and the inclusion of more OSA-related variables are also necessary to comprehensively evaluate the impact of OSA on children’s cognitive functions.

## 5. Conclusions

This study demonstrates that children with OSA generally maintain normal cognitive levels, as reflected by an average WISC-IV score of 102.32. However, some specific domains may be negatively affected, particularly in children with higher BMI scores. A significant negative correlation was observed between BMI scores and verbal comprehension, highlighting the role of obesity-related inflammation and metabolic changes in cognitive decline. While the OAHI showed a weak correlation with perceptual reasoning, it did not significantly impact the overall cognition. Factors such as tonsillar hypertrophy and gender had no measurable effect on the cognitive outcomes. Despite these findings, the study’s small sample size and absence of a control group limit its generalizability. Future research should adopt a larger, more diverse cohort and long-term follow-ups to better understand the multidimensional impact of OSA on children’s cognitive development. Managing BMI scores and early intervention remain critical for mitigating the specific cognitive risks associated with OSA.

## Figures and Tables

**Table 1:** Socio-demographic characteristics of the children (*n* = 110).

Variable	Frequency	Mean (SD)/%
Sex		
Male	70	63.6%
Female	40	36.4%
Anthropometric Variables		
A/N Ratio	110	1.32 ± 6.03
Tonsil Grading		
First degree	40	36.4%
Second degree	41	37.3%
Third degree	24	21.8%
Fourth degree	5	4.5%
BMI Z-score	110	0.04 ± 1.47
Body mass state		
underweight	15	13.6%
normal weight	65	59.1%
overweight	17	15.5%
obesity	13	11.8%
Polysomnographic Variables		
OAHI	110	9.77 ± 12.16
Lowest Blood Oxygen Saturation	110	86.41 ± 5.37
WISC-IV	110	102.32 ± 13.24

**Table 2:** Cognitive levels by gender.

	Male	Female	*t*	*p*
WISC-IV	103.29 ± 14.39	100.63 ± 10.92	1.014	0.313
VCI	100.63 ± 10.92	101.07 ± 17.47	1.092	0.278
PRI	101.07 ± 17.47	95.58 ± 17.92	1.573	0.119
WMI	95.58 ± 17.92	107.71 ± 14.77	1.562	0.122
PSI	107.71 ± 14.77	105.73 ± 14.77	0.679	0.498

**Table 3:** Levels of cognition under different tonsil gradings.

	First Degree	Second Degree	Third Degree	Fourth Degree	F	*p*
WISC-IV	96.69 ± 14.72	104.86 ± 10.77	104.13 ± 13.50	98.25 ± 12.80	1.196	0.315
VCI	92.54 ± 13.95	101.62 ± 15.07	100.89 ± 20.07	95.55 ± 14.88	1.084	0.359
PRI	103.23 ± 17.39	110.86 ± 10.50	107.52 ± 15.05	103.90 ± 15.68	0.689	0.561
WMI	93.77 ± 15.07	99.00 ± 10.34	98.91 ± 14.19	96.10 ± 12.49	0.370	0.775
PSI	97.77 ± 16.13	100.76 ± 11.42	116.71 ± 121.05	98.45 ± 12.19	2.075	0.108

**Table 4:** Correlation analysis of dimensions of cognitive level with other factors.

	WISC-IV	VCI	PRI	WMI	PSI
Age	−0.190 *	−0.018	−0.254 **	−0.170	−0.087
Time of illness	0.052	0.126	−0.093	−0.044	−0.081
Anthropometric Variables					
A/N Ratio	0.028	−0.049	0.015	0.040	0.133
BMI Z-score	−0.242 **	−0.204 **	−0.171	−0.124	−0.40
Polysomnographic Variables					
OAHI	−0.162	−0.085	−0.188 *	−0.168	0.050
Lowest Blood Oxygen Saturation	0.168	0.095	0.145	0.184	−0.001

*: *p* < 0.05; **: *p* < 0.01. Abbreviation explanations: VCI (Verbal Comprehension Index), PRI (Perceptual Reasoning Index), WMI (Working Memory Index), and PSI (Processing Speed Index).

**Table 5:** Multiple linear regression analysis of factors influencing WISC-IV scores.

Independent	B	β	*t*	*p*
BMI Z-score	−1.903	−0.211	−2.219	0.029

## Data Availability

The original contributions presented in the study are included in the article, further inquiries can be directed to the corresponding author.
